# Molecular characterization of vernalization loci VRN1 in wild and cultivated wheats

**DOI:** 10.1186/1471-2229-10-168

**Published:** 2010-08-11

**Authors:** Kseniya A Golovnina, Elena Ya Kondratenko, Alexander G Blinov, Nikolay P Goncharov

**Affiliations:** 1 Laboratory of Molecular-Genetic Systems, Institute of Cytology and Genetics, Novosibirsk 90, Russian Federation; 2 Laboratory of Wheat Genetics, Institute of Cytology and Genetics, Novosibirsk 90, Russian Federation

## Abstract

**Background:**

Variability of the *VRN1 *promoter region of the unique collection of spring polyploid and wild diploid wheat species together with diploid goatgrasses (donor of B and D genomes of polyploid wheats) were investigated. Accessions of wild diploid (*T. boeoticum*, *T. urartu*) and tetraploid (*T. araraticum, T. timopheevii*) species were studied for the first time.

**Results:**

Sequence analysis indicated great variability in the region from -62 to -221 nucleotide positions of the *VRN1 *promoter region. Different indels were found within this region in spring wheats. It was shown that *VRN1 *promoter region of B and G genome can also contain damages such as the insertion of the transposable element.

Some transcription factor recognition sites including hybrid C/G-box for TaFDL2 protein known as the *VRN1 *gene upregulator were predicted inside the variable region. It was shown that deletions leading to promoter damage occurred in diploid and polyploid species independently. DNA transposon insertions first occurred in polyploid species. At the same time, the duplication of the promoter region was observed in A genomes of polyploid species.

**Conclusions:**

We can conclude that supposed molecular mechanism of the *VRN1 *gene activating in cultivated diploid wheat species *T. monococcum *is common also for wild *T. boeoticum *and was inherited by *T. monococcum*. The spring polyploids are not related in their origin to spring diploids. The spring *T. urartu *and goatgrass accessions have another mechanism of flowering activation that is not connected with indels in *VRN1 *promoter region. All obtained data may be useful for detailed insight into origin of spring wheat forms in evolution and domestication process.

## Background

Some plants of middle latitudes require vernalization treatment (a long exposure to low temperatures) to induce their flowering. This adaptation appeared to protect flower from destruction by cold temperature during winter. Besides being an important trait for adaptation, the requirement of vernalization is also of a great agronomical importance. In crops such as common wheat (*Triticum aestivum *L.), a vernalization requirement distinguishes winter varieties from spring ones. Spring growth habit is a potential advantage in cold climates, with short vegetation season and cold winter, for agriculture which main task is to achieve rich harvest. In continental middle latitude areas (from 40° to 60° N latitude) the field is occupied for two vegetation seasons by winter forms and only for one season - by spring ones. So spring forms allow obtaining two harvests instead of one of winter growth habit forms, which, moreover, do not overwinter every year. Due to this reason, the processes underlining vernalization and genes responsible for this agronomical feature have been of interest for many years [1]. Increasing knowledge about the molecular genetics of growth habit (spring *vs*. winter) would contribute to a better understanding of the evaluation of adaptation in crops. This data would promote wheat breeding for specific environments.

In recent years, researchers made some breakthroughs in understanding of the molecular mechanisms of vernalization in wheat, a plant of prime agronomic importance. Sequences of three vernalization genes *VRN1, VRN2, VRN3 *responsible for spring-winter growth habit have been determine [2-5].

The predicted model shows that the *VRN1 *gene is dominant for spring growth habit and it is the main initiating factor of the flowering regulatory cascade that is upregulated by *VRN3 *(similar to *Arabidopsis FLOWERING LOCUS T, FT*) [6]. Whereas *VRN2 *gene is dominant for winter growth habit, it has been suggested that mediated protein [7]. In a complementary study, it was shown that nucleotide polymorphisms or insertions-deletions on A and D copies of the wheat *FT *gene can also explain variations for heading date [8].

Investigation of the photoperiod influence on the regulation of vernalization genes showed that down-regulation of the *VRN2 *by switching from LD (long day) to SD (short day) treatment has no effect on the regulation of *VRN1 *until plants are transferred again to LD [9]. These data suggest the existence of one more *VRN1 *repressor.

It has been demonstrated that functional difference between the dominant *Vrn1 *and recessive *vrn1 *alleles in cultivated diploid *T. monococcum *L. (genome A^b^A^b^) is due to deletions in the promoter region (near CArG-box) [3]. *Vrn-A1 *alleles of the common wheat (genome BBA^u^A^u^DD) and the tetraploid *T. turgidum *L. (genome BBA^u^A^u^) have also nucleotide deletions as well as insertions of mobile elements in *VRN1 *promoter and large deletions inside the first intron [10,11]. At the same time, the dominant *Vrn-B1 *and *Vrn-D1 *genes are caused only by large deletions in the first intron [11]. It is more likely that *VRN1 *promoter and the first intron regions contain the regulatory sites for different protein interactions. CArG-boxes are generally recognized by MADS-box genes and considered as the critical regulatory site for the response of *VRN1 *to SD. Recently, this hypothesis was refuted [12]. However, damages in either intron or promoter sites are sufficient to accelerate flowering under LD [10,11]. These data suggest the existence of interactions between these two mechanisms in the regulation of the *VRN1 *gene expression.

Most of the wild *Triticeae *Dum. species have a winter growth habit, suggesting that the recessive *vrn1 *allele is the ancestral feature. In contrast, the majority of cultivated polyploid wheats are spring species [13]. Only *T. aestivum *has the similar number of spring and winter forms http://www.vir.nw.ru and adaptability of landraces differed depending on their growth habit and *VRN *genotype [14]. Their growth habit is due to at least one dominant *Vrn1 *allele. This allele could be either inherited from ancestral diploids or could result from selection of independent mutations that appeared during the adaptation to various growing condition after domestication. Moreover, genetic and molecular experiments show that there are multiple alleles of dominant *Vrn1*, and their manifestation in vernalization response is also different [15,16]. It means that some mechanisms exist, for which evolutionary background is unknown. In the present study, molecular variability of wheat *VRN1 *promoter region was analyzed. In order to get a more detailed insight into complicated molecular basis of spring *vs*. winter growth habit and associated vernalization genes variability, we studied wild and cultivated wheats together with their close relatives *Aegilops *L. In total, 27 accessions of four diploid species (*T. urartu *Thum. ex Gandil., *T. boeoticum *Boiss., *T. monococcum *and *T. sinskajae *A. Filat. et. Kurk.) and 17 accessions of 7 polyploid species belonging to three known sections (*Dicoccoides *Flaksb., *Triticum*, *Timopheevii *A. Filat. et Dorof.) were investigated. Seven accessions of goatgrasses belonging to 2 species (*Aegilops squarossa *L., *Aegilops speltoides *Tausch), which participated in the hybridization events in polyploidy wheat evolution, were also studied. Among all 51 investigated accessions, eight have winter growth habit, and the remaining - spring growth habit. The number of genes responsible for spring *vs*. winter growth habit was estimated in the genetic experiments for ten spring accessions.

## Results

### Genetic analysis of growth habit (spring *vs*. winter)

For the molecular characterization, it was important to study accessions that possess monogenic control of spring growth habit. Genetic experiments were carried out to demonstrate genetic control of growth habit in some spring wheat and goatgrass species. The obtained data are given in Tables 1 and 2.

**Table 1:** F_2 _segregation for growth habit and tests of conformity to one and two gene ratios in crosses of *T. urartu *(u), *T. boeoticum *(b), *T. monococcum *(m), *T. sinskajae *(s) and *Ae. squarrosa *(ae)

Cross combination	**Segregation into winter *vs. *spring forms in the F**_**2 **_**generation**		**χ**^**2 **^value for ratio*	
	**spring**	**winter**	**3:1**	**15:1**

mKT3-5 × mPI306547	113	38	0,00	92,21

mPI306547 × bIG116198	14	4	0,07	7,84

bK25811 × bIG45296	96	30	0,10	66,31

bK-20741 × mPI306547	53	27	3,27	103,25

bIG116198 × mPI35526	57	13	1,54	18,14

sK-48993 × mPI13962	146	55	0,60	152,92

aeK-992 × aeK-608	37	0	-	-

aeK-865 × aeKU2009	117	0	-	-

aeKU2009 × aeKT120-16	197	50	2,98	82,54

* - values for significance of P = 0.05 is 3.84 and P = 0.01 is 5.99; spring accessions are underlined

**Table 2:** Calculation of number of *VRN *genes in tetraploid wheat species

Species, accessions	**Segregation into spring *vs*. winter forms in the F**_**2 **_**generation**			Genotype (Haploid)
	
	BWE (*vrn-A1*)	BS1E (*Vrn-A1*)	BS2E (*Vrn-B1*)	
*T. durum cv. *Kristall **(***Vrn-D4*)	50:16^1)^	116:13^2)^	122:6^2)^	*Vrn-D4*

*T. turgidum *K-16156	187:57^1)^	60:5^2)^	28:3^2)^	*vrn-A1vrn-B1Vrn-Т*

*T. turanicum *K-15993	-	157:10^2)^	69:7^2)^	*vrn-A1vrn-B1Vrn-T*

BWE - cv. *T. dicoccum *Black Winter Emmer, i: BS1E - Black Spring *Vrn-A1*

Emmer, i: BS2E - Black Spring *Vrn-B1 *Emmer, Kristall(*Vrn-D4*) - introgressive spring line of *T. durum *winter cv. Kristall with dominant gene *Vrn-D4*.

1)	- monogenic control, χ^2 ^value for ratio is not higher than 3.84

2)	- digenic control, χ^2 ^value for ratio is not higher than 3.84

It was established that spring growth habit of most of examined species was controlled by a single dominant gene. In the Table 1 the segregation ratio 3 to 1 is reliable while the value of Pierson chi-square test for the segregation ratio 15 to 1 is higher than the value of significance 3.84. Three investigated tetraploid species were crossed with near-isogenic test lines, which were characterized with specific dominant *Vrn-A1 *(i: BS1E), *Vrn-B1 *(i: BS2E) alleles. The design of near-isogenic lines is described in Methods section. In crosses with *T. dicoccum *cv. Black Winter Emmer (BWE), which has recessive *vrn-A1 *and *vrn-B1 *alleles, it was shown that spring growth habit of these species was controlled monogenic, because the segregation ratio 3 to 1 in F_2 _generation is reliable. For *T. turanicum *(K-15993) monogenic control was shown indirectly from the next two crosses, where the digenic segregation (15 to 1) was obtained for all three species. It means that dominant allele responsible to spring growth habit is not allelic to *Vrn-A1 *and *Vrn-B1 *alleles of the tested lines.

In tetraploid wheat species, the dominant gene of the accessions K-15993 and K-16156 was temporarily designated as *Vrn-T *and their genotypes were specified as *vrn-A1vrn-A1 vrn-B1vrn-B1 VrnTVrnT*. It is an unknown allele, which is nonallelic to *Vrn-A1 *and to *Vrn-B1*. The number and allelity of dominant genes *VRN1 *in *T. timopheevii *(Zhuk) Zhuk. was not determined because all accessions of this species had only spring growth habit [13]. The generalized information concerning genetic control of growth habit is represented in Table 3.

**Table 3:** 

Genus, section, genome, species	Accession-voucher, cultivars	Gr. habit	Genotype or number of dominant genes*	*VRN1 *allele**	GenBank Ac.N.
Triticum L. section *Urartu *Dorof. et A.Filat. *A^u^A^u^* *T. urartu *Thum. ex Gandil.					

	K-33869	W		*vrn-A1*	GQ451714

	PI538736	W		*vrn-A1*	GQ451724

	PI428297	S	unknown	*vrn-A1*	GQ451721

	PI428197	S	unknown	*vrn-A1*	GQ451723

	IG44829	S	unknown	*vrn-A1u*	GQ451737

	IG45298	S	unknown	*vrn-A1*	GQ451736

section *Monococcon *Dum. *A^b^A^b^* *T. monococcum *L.					

	PI94743	W		*vrn-A1*	GQ451733

	K-20400	S	mono[44]	*vrn-A1*	GQ451715

	K-18105	S	unknown	*Vrn-A1g*	GQ451717

	Mute KT3-5	S	mono (Table1)	*vrn-A1*	GQ451725

	K-20970	S	unknown	*vrn-A1*	GQ451730

	PI306540	S	unknown	*vrn-A1*	GQ451732

*T. boeoticum *Boiss.					

	G1777	W		*vrn-A1*	GQ451718

	K-25811	W		*vrn-A1*	GQ451728

	IG116198	S	mono (Table1)	*Vrn-A1h*	GQ451727

	K-14384	S	unknown	*vrn-A1*	GQ451716

	PI428217	S	unknown	*vrn-A1*	GQ451719

	IG116196	S	mono (Table1)	*vrn-A1*	GQ451720

	PI427328	S	unknown	*Vrn-A1h*	GQ451722

	K-20741	S	mono (Table1)	*Vrn-A1h*	GQ451734

	IG45296	S	mono (Table1)	*Vrn-A1h*	GQ451735

	K-40117	S	unknown	*Vrn-A1g*	GQ451738

	K-40118	S	unknown	*Vrn-A1h*	GQ451739

	KU8136	S	unknown	*Vrn-A1h*	GQ451743

	KU8120	S	unknown	*Vrn-A1h*	GQ451744

	KU8059	S	unknown	*Vrn-A1h*	GQ451745

*T. sinskajae *A. Filat. et Kurk.					

	K-48993	S	[45] mono (Table1)	*vrn-A1*	GQ451729

section *Dicoccoides *Flaksb. *BBA^u^A^u^* *T. turgidum *L.					

	K-16156	S	*Vrn-T *(Table 2)	*vrn-A1u* *vrn-B1*	GQ451819-GQ451820

*T. durum *Desf.					

	spring line cv.Kristall	S	*Vrn-D4 *(Table 2)	*Vrn-A1e* *vrn-B1*	GQ451821-GQ451822

*T. dicoccum *(Schrank) Schuebl.					

	i: BS1E	S	*Vrn-A1 *[36]	*Vrn-A1a* *vrn-B1^3^*	GQ451756-GQ451759

	i: BS2E	S	*Vrn-B1 *[36]	*Vrn-A1b* *vrn-B1*	GQ451760-GQ451761

*T. turanicum *Jakubz.					

	K-15993	S	*Vrn-T *(Table 2)	*Vrn-A1f^1^* *vrn-A1u^2^* *vrn-B1*	GQ451815-GQ451818

	K-11597	S	unknown	*Vrn-A1b^1^* *vrn-A1u^1^* *Vrn-B1a^2^* *vrn-B1^1^*	GQ451768-GQ451772

section *Triticum* *BBA^u^A^u^DD* *T. aestivum L.*					

	cv. Mironovskaya yubileinaya	W		*vrn-A1u* *vrn-B1* *vrn-D1*	GQ451781-GQ451783

	cv. Mironovskaya 808	W		*vrn-A1u* *vrn-B1* *vrn-D1^2^*	GQ451784-GQ451787

	nulli5B-tetra5 D Chinese Spring	S	*Vrn-D1 *(4 dose) [46]	*Vrn-A1f^2^* *vrn-A1u^2^* *vrn-D1^4^*	GQ451773-GQ451780

	cv. Pyrothrix 28	S	*Vrn-A1b*, *VrnB1 *[15]	*vrn-A1u^3^* *vrn-B1* *vrn-D1^2^*	GQ451788-GQ451793

	cv. Jupateko	S	*Vrn1 *(weak) [47]	*Vrn-A1f^1^* *Vrn-A1a^1^* *vrn-A1u^1^* *vrn-B1* *vrn-D1*	GQ451794-GQ451798

	s: Saratovskaya/Vietnamskaya 5R(5A)	S	unknown	*Vrn-A1b^3^* *vrn-B1* *vrn-D1*	GQ451799-GQ451803, GQ451814

	cv. Mironovskaya yarovaya	S	*Vrn1*[48]	*Vrn-A1f^1^* *vrn-A1u^3^* *vrn-B1* *vrn-D1^2^*	GQ451804-GQ451809

	cv. Osijek	S	*Vrn-A1b *[47]	*vrn-A1u, vrn-B1* *vrn-D1^2^*	GQ451810-GQ451813

section *Timopheevii *A. Filat. et Dorof. *GGA^b^A^b^* *T. araraticum *Jakubz.					

	K-28244	S	unknown	*Vrn-A1f^4^* *vrn-G1^2^*	GQ451762-GQ451767

*T. timopheevii *(Zhuk) Zhuk.					

	K-38555	S	unknown	*Vrn-A1f^4^* *vrn-A1u^1^* *Vrn-G1a*	GQ451750-GQ451755

Tetraploid line					

	PI428276	S	unknown	*Vrn-A1g^1^* *Vrn-A1f^2^* *vrn-A1u^3^* *vrn-A1^1^*	GQ482969-GQ482975

Aegilops L. section *Sitopsis *(Jaub. & Spach) Zhuk. *SS* *Ae. speltoides *Tausch					

	Ae46593	W		*vrn-S*	GQ451726

	Ae46566	S	unknown	*vrn-S*	GQ451741

section *Vertebrata *Zhuk. emend. Kihara *DD* *Ae. squarrosa *L.					

	K-992	S	di [31]	*vrn-D1*	GQ451731

	K-865	S	mono [31]	*vrn-D1*	GQ451740

	K-608	S	mono [31]	*vrn-D1*	GQ451742

	K-864	S	mono [31]	*vrn-D1*	GQ451746

	KU2009	S	mono (Table 1)	*vrn-D1^3^*	GQ451747-GQ451749

### Allelic variability at the *VRN1 *promoter region of diploid species

#### Variability at the primer annealing site

**Figure 1:**
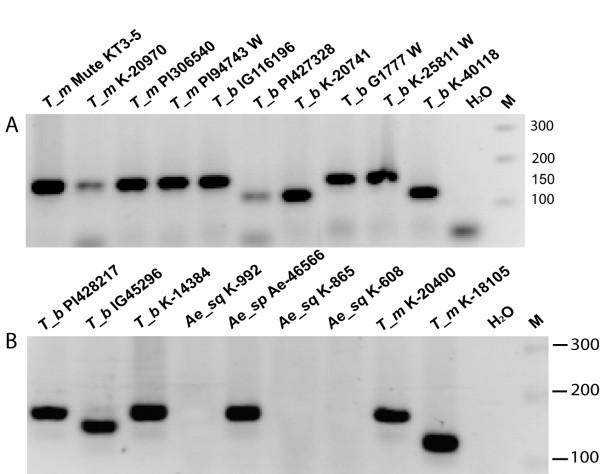
**PCR amplification of *VRN1 *promoter region with primers *AP1_ProDel_F1/AP1_ProDel_R1***. A. Diploid wheat accessions. T_b - *T. boeoticum*, T_m - *T. monococcum*, Ae_sp - *Ae. speltoides*, Ae_sq - *Ae. squarrosa*. T_b PI-427328, K-20741, K-40118 have 20 bp deletion. W - winter growth habit. B. Spring diploid *Triticum *and *Aegilops *accessions. T_b IG-45296 has 20 bp deletion, T_m K-18105 has 34 bp deletion. M - Marker-Bioline HyperLader IV.

In the present work, 27 diploid wheat accessions of four known species (*T. urartu*: winter - K-33869, PI538736; spring - PI428297, PI428197, IG44829, IG45298; *T. monococcum*: winter - PI94743; spring - K-20400, K-18105, Mute KT3-5, K-20970, PI306540; *T. boeoticum*: winter - G1777, K-25811; spring - IG116198, K-14384, PI428217, IG116196, PI427328, K-20741, IG45296, K-40117, K-40118, KU8136, KU8120, KU8059; *T. sinskajae*: spring - K-48993) and 7 goatgrass accessions of two species (*Ae. speltoides*: winter - Ae46593, spring - Ae46566; *Ae. squarrosa*: spring - K-992, K-865, K-608, K-864, KU2009) were studied (see Material section and Table 3). Initial PCR screening was provided with primer pair AP1_ProDel_F1, AP1_ProDel_R1 (Table 4). The PCR product of the expected size of 152 bp has been obtained in the majority of the studied wheat accessions and in one goatgrass species, *Ae. speltoides *(Fig. 1). No PCR products have been found in *Ae. squarrosa *accessions. Out of 27 wheat accessions, ten showed PCR products of the lower size, which can be explained by deletions in the promoter region.

**Table 4:** Primer pairs used in the study

Name	Sequence	Reference
AP1_ProDel_F1	5'ACAGCGGCTATGCTCCAG3'	[3]
AP1_ProDel_R1	5'TATCAGGTGGTTGGGTGAGG3'	[3]

AP1_2F	5'CTGTGGTGTGTGTTTGTGGCGAGAG3'	Present study
AP1_2R	5'ACCCTACGCCCCTACCCTCCAACAC3'	

VRN1-AF	5'GAAAGGAAAAATTCTGCTCG3'	[10]
VRN1-BF	5'CAGTACCCCTGCTACCAGTG3'	[10]
VRN-DF	5'CGACCCGGGCGGCACGAGTG3'	[10]
VRN1-1NT1R	5'TGCACCTTCCC(C/G)CGCCCCAT3'	[10]

**Figure 2:**
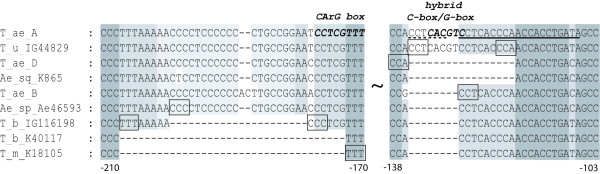
**Deletions observed in the *VRN1 *promoter alleles among spring diploid species**. Nucleotide positions are indicated according to *T. aestivum vrn-A1 *(GenBank: AY616455) promoter. In boxes there were represented 3 letter repeats flanked the place of the deletions. The *AP1_ProDel_R1 *primer annealing site is indicated by solid line and 6 bp repeat near this region in A genome - by dashed line. T_m -*T. monococcum*, T_u - *T. urartu*, T_b - *T. boeoticum*, T_ae - *T. aestivum*, Ae_sp - *Ae. speltoides*, Ae_sq - *Ae. squarrosa*. T_ae_TDC A*- vrn-A1 *(AY616455), T_ae_TDC B- *vrn-B1 *(AY616456), T_ae_TDC D- *vrn-D1 *(AY616457) were obtained from GenBank.

The heterogeneity of diploid genomes and their sequence variability was observed previously [17,18]. All available *VRN1 *promoter sequences belonging to different wheat genomes (A, B, D) were extracted from GenBank and aligned together with primer sequences. There were a 17 bp deletion in D genome near the region complementary to the reverse primer (AP1_ProDel_R1), and a duplicated fragment (CCTCAC) near this region in A genome (Fig. 2). Therefore, a new primer pair was developed (AP1_2F/AP1_2R) for amplification of D genome. To check for possible minor deviations connected with spring growth habit, all fragments obtained in PCR using both primer pairs were directly sequenced. The resulting sequences were submitted to GenBank: GQ451714-GQ451749.

#### The presence of the specific deletions in spring wild and cultivated accessions

Among 22 investigated spring diploid wheats, 9 (*T. monococcum*: K-18105, *T. boeoticum*: K-40117, K-40118, PI427328, K-20741, IG45296, KU8136, KU8120, KU8059) had 20 bp or 34 bp deletions in *VRN1 *promoter region comparing with winter ones (Table 3, Fig. 2). Both deletions covered the specific area of MADS-box, and were previously described as associated with spring growth habit in the diploid cultivated wheat *T. monococcum *[3,4]. Spring *T. boeoticum *accessions were not screened previously as well as *T. urartu*. Investigation of the *VRN1 *promoter mutations in both species may provide important information about mechanisms involved in the origin of the spring forms in these wild natural species. Among four spring *T. urartu *accessions, none had specific deletions within the *VRN1 *promoter region compared to winter forms. However, one accession was almost identical to the A genome sequences of the polyploid wheats (Fig. 2). This recessive allele was named *vrn-A1u *and was not found in all analyzed *T. boeoticum *and *T. monococcum *sequences (Table 3).

The closest relatives of *Triticum *L. species are *Aegilops *L. species, which also have spring and winter forms. In order to address the question whether flowering initialization in spring goatgrasses has common characters with that of diploid wheats, we obtained *VRN1 *promoter regions from six spring and one winter *Aegilops *accessions. There were some nucleotide substitutions differentiating goatgrasses from wheats (see additional file 1). No significant changes, which could distinguish spring and winter accessions, have been found. All investigated promoter sequences of the *Ae. squarrosa *accessions contained the specific D genome 17 bp deletion (Fig. 2) and some D genome specific nucleotide substitutions.

### Allelic variation at the *VRN1 *promoter region in polyploid wheats

#### Tetraploid wheat species

**Figure 3:**
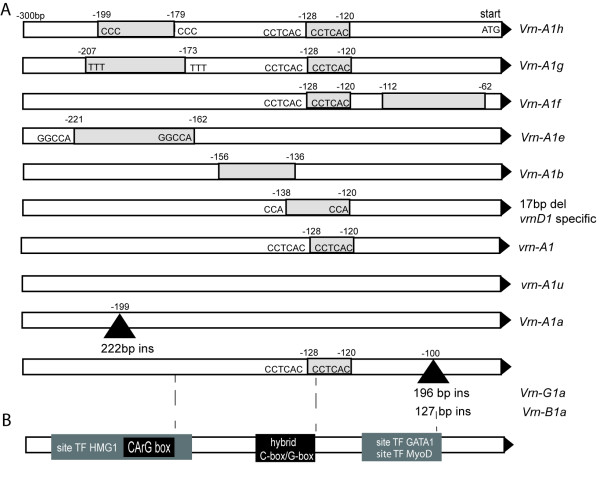
**Indels and regulatory sites found in *VRN1 *promoter region**. A. Schematic representation of the different *VRN1 *promoter variants observed in spring wheat accessions. Places of indels are marked in bp. numbers upstream from the start codon. Grey boxes indicate deletions and black triangles - insertions. One of the direct repeats flanked deletions that remain in sequence after recombination is outside the grey box. B. The predicted transcription factors binding sites.

**Figure 4:**
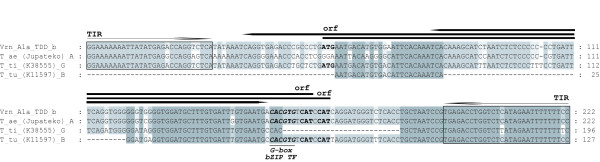
**Alignment of foldback elements insertion observed in different *VRN1 *alleles**. T_ae-*T. aestivum*, T_ti-*T. timopheevii*, T_tu-*T. turgidum*, TDD-*T. aestivum *(near-isogenic line Triple Dirk D).

*VRN1 *promoter regions from G genomes of spring *T. timopheevii *(K-38555) and *T. araraticum *Jakubz. (K-28244) were amplified using primer specific for B genome. We analyzed also a tetraploid line determined by karyological analysis (PI428276), which was found in an accession of *T. urartu *from Lebanon. Sequencing showed a close relatedness of the obtained G genome to the corresponding B genome (see additional file 2). The most interesting was the presence in *T. timopheevii *G genome and two clones of *T. turanicum *Jakubz. B genome of several identical substitutions and large insertions in -100 position upstream from the start codon. The insertions were 196 bp long in G genome and 127 bp in B genome (Fig. 3). We named these *VRN1 *promoter variants *Vrn-G1a *and *Vrn-B1a*. These insertions were similar to a 222 bp foldback element (GenBank: AY616458) observed in *Vrn-A1a *allele and named "Spring" (Fig. 4). However, the site of insertion was different. It was not in the place of the CArG box (CCTCGTTTTGG) and was flanked by another host direct duplication (HDD - CTCCGCCCC) (Fig. 3).

More detailed analysis of the mobile elements which were found in *Vrn-A1*, *Vrn-B1 *and *Vrn-G1 *promoter regions of tetra- and hexaploid wheat species showed the presence of the terminal inverted repeats (TIR) and predicted open reading frames between both repeats (Fig. 4). The observed transposon element, which is 222 bp in length with 29 bp TIR, could belong to the superfamily Tc1/Mariner.

The *VRN1*promoter regions from A genomes of spring tetraploids also contain different promoter damages and share some variability. In addition, a 50 bp deletion was found in the region -112 and -62 nucleotide from start codon. This deletion was located in the same region where the foldback element insertion occurred in the G genome (Fig. 3). This *VRN1 *promoter allele was called *Vrn-A1f*. Species of the section *Dicoccoides*, *T. turanicum *(K-11597) and *T. dicoccum *(Schrank) Schuebl. (line BS2E) also had a common 20 bp deletion in the region -156 -136 upstream from the start codon. This deletion included the repetitive region and was observed previously in *Vrn-A1b *and *Vrn-A1d *alleles of spring tetraploid *T. durum *Desf. and *T. dicoccoides *correspondingly [10]. We observed also a 54 bp deletion in A genome of *T. durum *(spring line of cv. Kristall (*Vrn-D4*), Odessa) that was similar to *Vrn-A1e *alleles of *T. dicoccum*. *VRN1 *promoter from A genome of *T. dicoccum *(near-isogenic line BS1E - Black Spring *Vrn1 *Emmer) displayed identity with *Vrn-A1a *allele observed previously in *T. aestivum *near-isogenic line Triple Dirk D (TDD) and contained the insertion of a foldback element.

#### Hexaploid wheat species

Promoter sequences of *vrn-A1*, *vrn-B1 *and *vrn-D1 *alleles from two winter cultivars (Mironovskaya yubileinaya, Mironovskaya 808) and A, B and D genome sequences of spring mute Mironovskaya yarovaya were determined. The spring mute was found in cultivar Mironovskaya 808. *VRN1 *promoter fragments of winter cultivars and B, D genome of spring Mironovskaya yarovaya contained a common for certain genomes variability observed in recessive alleles of winter wheat near-isogenic line Triple Dirk C (TDC) [10]. After cloning and sequencing of A genome fragment of spring sample, clones with and without specific deletions were observed. The observed deletions included 8 bp del in -128 -120 region and 50 bp del in -112 -62 region that were common for the *Vrn-A1f *allele described above in tetraploid species (Table 3, Fig. 3).

Three *T. aestivum *cultivars Pyrothrix 28, Osijek and Jupateko with "weak" *Vrn1 *alleles (low phenotype manifestation allele) [15] were also included in the research. No differences from recessive alleles were found in *VRN1 *promoter sequences from A, B and D genomes of spring cvs. Osijek and Pyrothrix 28; as well as from B and D genomes of spring cv. Jupateko. After amplification of the *VRN1 *promoter region from A genome of the cv. Jupateko, two PCR products were visualized on electrophoresis. Sequencing of two larger PCR products revealed the presence of the foldback element insertions that was common for *VrnA1-a *allele [10]. A 222 bp foldback element insertion differed moderately from those previously described. The site of insertion and flanking direct repeats (TTAAAAACC) were similar for both elements. However, analysis of the foldback element of spring cv. Jupateko indicated an absence of direct repeats inside (Fig. 4). These direct repeats were the sites of deletions in the duplicated promoter sequences of *Vrn-A1a *allele. Therefore, we detected only one variant of the promoter with mobile element insertion in this species. The smaller PCR products in cv. Jupateko had most common character with *Vrn-A1f *allele observed in K-38555 *T. timopheevii*. Thus, two different *Vrn-A1 *alleles were detected. Both alleles could be the cause of spring growth habit.

We also found promoter damages in the A genomes of two other investigated intrageneric *T. aestivum*: substitution line Saratovskaya/Vietnamskaya 5R(5A) and line nulli5B-tetra5D Chinese Spring. The investigated promoter regions contained *Vrn-A1b *and *Vrn-A1f *alleles correspondingly (Table 3). Both alleles could be a reason of spring growth habit and likely inherited from tetraploid species during cultivation and breeding programs.

#### Variability of different clones inside A genome

**Figure 5:**
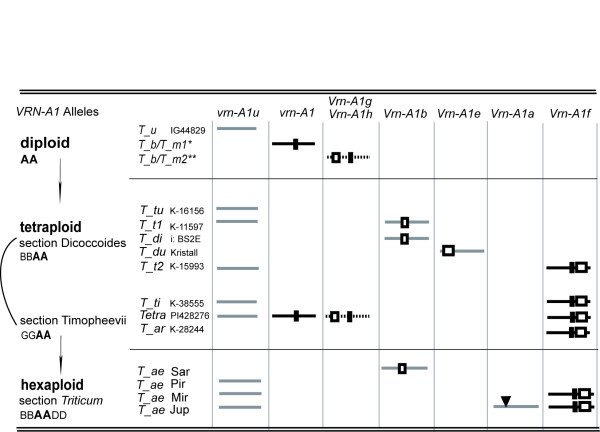
**Variable *VRN-A1 *promoter copies**. Boxes depict deletions (small black box -8 bp deletion), triangles - transposon insertion. *T_m -T. monococcum, T_u - T. urartu , T_b - T. boeoticum, T_tu - T. turgidum, T_di - T. dicoccum, T_du - T. durum, T_t - T. turanicum, T_ti - T. timopheevii, T_ar - T. araraticum, T_ae *Sar - *T. aestivum *s: Saratovskaya/Vietnamskaya 5R(5A), *T_ae *Pir - *T. aestivum *cv. Pyrothrix 28, *T_ae *Mir - *T. aestivum *cv. Mironovskaya yarovaya, *T_ae *Jup - *T. aestivum *cv. Jupateko. *T_b/T_m *1,2 and *T_t *1,2 show two different allele's variants between accessions of indicated species. Accession number: * - T. m. winter -PI94743, spring - K-20400, Mute KT3-5, K-20970, PI306540; T. b. winter - G1777, K-25811, spring - K-14384, PI428217, IG116196. ** - T. m. spring - K-18105 (*Vrn-A1g*); T. b. spring - IG116198, PI427328, K-20741, IG45296, K-40118, KU8136, KU8120, KU8059 (*Vrn-A1h*), K-40117 (*Vrn-A1g*).

Investigation of A genome sequences revealed at least two different variants of *VRN1 *promoter in some cloned samples according to the presence of indels. At the same time all cloned B and D genome sequences were identical in the presence or absence of indels. To exclude any doubt in affiliation with a specific genome, we provided the phylogenetic analysis, which allowed the correlation of each sequence into the genome (see additional file 3). Each of three accessions of *Diccoccoides *section (*T. turanicum *K-11597, K-15993; *T. dicoccum *line BS1E) had two different variants of the promoter in A genome. One of them was intact as in winter *vrn-A1 *allele, and another had a deletion or insertion of the transposon (Fig. 5). *VRN1 *promoters of the remaining three examined species (*T. dicoccum *line BS2E, *T. durum *line Kristall (*Vrn-D4*), *T. turgidum *K-16156) were sequenced directly without cloning and contain only one variant of sequence judging from the absence of superpositions in the chromatograms.

In section *Timopheevii *four clones of *T. araraticum *(K-28244) were similar if indels are taken into consideration. *T. timopheevii *(K-38555) had four clones similar to those of *T. araraticum *(K-28244) containing *Vrn-A1f *allele and one clone with intact *vrn-A1 *allele. Absence of the previously determined A genome specific 8 bp insertion in the region -120 -128 were observed in some clones of the tetraploid species. Only A genome had variability at this region while B, G and D genomes as well as genomes of the examined goatgrasses were conservative and had deletions in this place. All obtained *Vrn-A1f *allele had no 8 bp insertion.

Seven clones of *VRN1 *promoter region from A genome were sequenced in tetraploid line discovered in *T. urartu *collection under Ac.N.PI428276 and later was determined as tetraploid according to karyological and molecular analyses. Four different promoter variants were found. At the same time, no positive result was obtained in PCR with B/G genome specific primers for *VRN1 *promoter region and developed previously for *Acc-1 *and *Pgk-1 *genes [18].

Three spring cultivars of hexaploid *T. aestivum *also contain at least two *VRN1 *promoter variants in A genomes (Fig. 5). *VRN1 *promoter region of A genome of cv. Mironovskaya yarovaya and line nulli5B-tetra5D Chinese Spring included the recessive *vrn-A1 *and the dominant *Vrn-A1f *alleles. Cultivar Jupateko had in addition the third allele differed from others by a transposon insertion.

### Predicted regulatory elements inside *VRN1 *promoter region

In the present work, we performed the analysis of the cis-regulatory elements (recognition sites for transcription factors, TF) inside the most variable region of the *VRN1 *promoter. This information helps to investigate the consequences of the damages in the promoter of the main initiation factor of flowering, and possibly is related to the mechanism of growth habit changing.

Previously, a specific CArG-box common for all MADS-box protein family was found in the region where different in size deletions and transposon insertion occurred in spring diploid and polyploid wheat species. It was suggested that CArG-box could be involved in the regulation of VRN1 by interacting with other MADS-box proteins [3]. Our analysis showed that this region also included recognition site for HMG1, a high mobility globular protein, with a high probability (error type I -0.2308, error type II - 0,03882, psum -91, 074, Fig. 3B). Recently, an increased expression of wheat homolog HMG1B protein after cold exposure of crown tissue was showed [19]. The HMGB1 is thought to interact with histones and could participate in modifying chromatin structure.

Some other regulatory sites were found in the region of 20 bp and 50 bp deletions common for *Vrn-A1b *and *Vrn-A1f *alleles, correspondingly (Fig. 3B). We also observed the recognition site (G/C hybrid box) for bZIP proteins. The VRN3 (TaFT) protein was shown to upregulate *VRN1 *expression via interaction with TaFDL members of bZIP transcription factor family [6,20]. Moreover, it appears that TaFDL2 protein can interact *in vitro *with ACGT elements of the promoter of the meristem identity gene *VRN1*, including G-box and G/C hybrid of G- and C-boxes (**C**ACGT**C**). It is interesting that both transposon insertions observed in B and G genomes contain additional G-box motif (CACGT**G**) (Fig. 4).

Near the region of the G/C hybrid box we found the binding motifs for MyoD-like protein, a member of the bHLH family of transcription factors (error type I -1, error type II - 5,2e-05, psum -94,4257) (Fig. 3B). Members of the bHLH family in animals have been shown to regulate the determination and differentiation of a variety of cell types, including skeletal muscle, neurons, and hematopoietic cells. Recent detailed study into the physical interaction among the bHLH proteins of *Arabidopsis *that control stomatal development reflected that a similar mechanism functions in animal muscle and plant stomata [21,22]. It is known also that markers of myogenic specification such as *MyoD *and myogenin gene, which encode transcription factors of the basic helix-loop-helix family, interact with proteins belonging to the MADS-box transcription factors [23]. It is likely that examined bHLH proteins (myoD-like) modulate cell cycle regulatory protein activity in a similar fashion in both animal and plant cells.

A binding motif was also found for GATA1 protein (error type I -0,1111, error type II -0,00451, psum -90,1055) (Fig. 3B). GATA transcription factors are a group of DNA binding proteins broadly distributed in eukaryotes. Four classes of these proteins are described in plants [24]. Some GATA transcription factors are downstream effectors of floral homeotic gene action in *Arabidopsis *[25]. It appears that two MADS-box gene products, APETALA3 (AP3) and PISTILLATA (PI) can directly interact with two members of GATA transcription factor family. Altogether they regulate a variety of light and nutrient responsive genes participating into the transcriptional cascades controlling the specification of floral organ identities. In particular, *APETALA1 *gene (homolog of *VRN1 *gene in wheat) is directly regulated by AP3 and PI in *Arabidopsis *[26].

Transcription factor binding motifs described here are found in the *VRN1 *promoter region where indels could induce flowering without cold treatment. This could indicate a possibility that wheat homologs of the HMG1, bZIP, bHLH and GATA1 proteins are involved in the vernalization response. Further experimental approaches are necessary to support this suggestion.

## Discussion

### *Triticum *genetic material

Notwithstanding the short evolutionary time since the domestication, some significant morphological changes were accumulated in cultivated plants. As a result of an intensive cultivation, many wheat varieties with different agronomical important characteristics were selected. However, in many cases the molecular pathway behind the appearance of a sample with an unusual character in the stable population is still unknown.

Winter growth habit is assumed to be a more ancestral character than the spring one. It is not known whether the ancient farmers found spring forms in nature or in their plots. However, they started to cultivate spring forms intensively. Among the first wheat species cultivated by people, *T. monococcum *and *T. dicoccum*, most have spring forms according to the last investigation of 284 and 563 accessions correspondingly [13]. The winter forms came back to occupy considerable areas in the middle latitudes at the beginning of the 20^th ^century. But in the continental middle latitude areas (countries like Kazakhstan, Russia, Ukraine, Belorussia, Canada) the field is occupied by winter forms for two vegetation seasons and by spring forms - for one season only. This is due to the agronomical aspects of spring forms - sowing in spring when winter is too cold. In these countries it is impossible to use the intermediate forms, which are used in Southern Europe and Mediterranean region, because of climate, when spring crop can not survive in winter. It appears that, when both forms are used, winter ones are often less economically beneficial. For this purpose, mutants with spring alleles of *VRN1 *in different genomes (A, В, D) were developed. Multiple alleles have been described within the VRN1 locus, based on their different effects on vernalization requirement and flowering time [15,16,27,28]. However, characterization of these alleles at the molecular level is necessary. The number of dominant *VRN *genes in common wheat is important since it modifies spring growth habit, and allows for the development of commercial cultivars with different vegetative periods.

Studying the unique collection of spring wild and domesticated diploid and polyploid wheats not examined before allowed us to observe a greater variability, especially in tetraploid and hexaploid species. We described three additional *VRN1 *promoter variants *Vrn-A1f*, *Vrn-A1g *and *Vrn-A1h*. Moreover, we showed that *VRN1 *promoter region of B and G genomes can also contain damages such as the insertion of a transposable element. The duplication of the *VRN1 *promoter together with the first exon was also demonstrated.

### Inheritance of *VRN1 *gene from spring wild diploids to cultivated diploids

It is known that wild diploid wheats were the donors of A genome in polyploid wheat species. Diploid species are the most ancient in the *Triticum *genus so their genome (A genome) has the longest history in the genome composition of the modern wheat species. The majority of the wild wheats have a winter growth habit, suggesting that the recessive *vrn-A1 *allele with an intact *VRN1 *promoter is the ancestral character. We studied the number of wild and cultivated diploid species, and established that *VRN1 *promoter deletions that resulted in the elimination of repressor influence in cultivated species *T. monococcum *are also common for wild species *T. boeoticum*. Therefore, it is possible to conclude that spring forms originated in wild populations and later were selected by man for culture. New alleles may result from selection of independent mutations that appeared during the domestication.

It is likely that a shorter 20 bp deletion appeared first and then increased to 34 bp deletion. Both variants were inherited by cultivated diploid forms and could be inherited also by polyploids.

Species of the genus *Aegilops *are closely related to wheats; they have common geographic range, and about 0.5 million years ago a first polyploid wheat appeared as a result of hybridization of wheats with goatgrasses [29]. Similar to wild diploid spring *Triticum *species, there are early-flowering forms among *Aegilops *species, which evolved in two intraspecific lineages, Middle Asian and Transcaucasian [30]. The existence of longitudinal and latitudinal clines in the onset of flowering at the species level was observed; however, mechanism controlling switch from vegetative to generative growth habit is unknown. To answer the question whether these mechanisms have common characters with that of diploid wheats we analyzed *VRN1 *promoter regions of several *Aegilops *accessions. Spring *Ae. squarrosa *accessions originated from the northern part of Afghanistan and Iran while in other regions only winter forms were usually found [31]. However, no indel events were found inside the investigated region. Therefore, taking into account the genealogical and ecological framework complexity of flowering time diversification in the investigated *Aegilops *accessions we expect the existence of another regulatory mechanism controlling flowering time in goatgrass species.

### Increasing genetic variability in spring tetraploids within A, B, and G genomes

In contrast with wild *Triticum *species, the majority of cultivated polyploid wheat species have spring growth habit [13]. Spring forms are due to at least one dominant mutation in *VRN1 *gene that could be inherited or appeared independently during domestication or historical selection process.

Four different variants of *VRN1 *promoter in A genomes of diploid species were observed (Fig. 5). The main difference between sequences is the presence or absence of a specific 8 bp deletion that was not observed only in one spring accession of *T. urartu *and inherited by A genome of many polyploids.

In comparison with diploid species three other new *VRN1 *promoter variants due to deletions appeared in tetraploids. One of them represents *Vrn-A1e *allele and contains 54 bp deletion in place where specific 20 and 34 bp deletions occurred in diploids. Possibly there was a deletion expansion in tetraploids but independent appearance is also likely due to the existence of flanking direct repeats, which may be the reason of nonhomologous recombination (Fig. 3). There are many different repeat regions inside *VRN1 *promoter and most of the deletions flanked by short direct repeats (Fig. 3). It was shown that unequal recombination by such repeats can play the main role in the origin of novel alleles in *VRN1 *gene [32]. The *Vrn-A1e *allele could have originated from the allele containing 8 bp insertion and observed only in one wild diploid species *T. urartu*.

Another *Vrn-A1b *allele found in tetraploids contains a 20 bp deletion (Fig. 3). Its origin is also related to *T. urartu VRN1 *promoter containing 8 bp insertion (Fig. 5). The described deletion most likely happened in species of *Dicoccoides *section. This allele is not found in wheat species from *Monococcon *Dum. and *Timopheevii *sections. At the same time, *Vrn-A1f *allele likely occurred in *Timopheevii *section within species with G genomes (Fig. 5). Moreover, its origin is related to diploid *VRN1 *promoter containing 8 bp deletion. We found this allele also in *T. turanicum *(section *Diccocoides*); this, however, is likely a case of hybridization event that may occur between tetraploid species from different wheat sections.

The *VRN1 *promoter variant found in *T. dicoccum *accession (near-isogenic line BS1E) had all common characters of *Vrn-A1a *allele as well as the transposon insertion (Fig. 3). Taking into consideration the origin of BS1E line, as the near-isogenic line of cv. Black Winter Emmer whose *vrn-A1 *allele was replaced with *Vrn-A1a *of hexaploid wheat Triple Dirk (TDD) by backcrossing, this proves its derivation from *T. aestivum *near-isogenic line TDD.

We found and described also two different alleles in B and G genomes of tetraploid species that contain a transposon insertion (*Vrn-G1a*, *Vrn-B1a*, Fig. 4). The indel events in these genomes were observed for the first time, and both species contained also *VRN1 *promoter damages in A genomes. Investigated hexaploid accessions of section *Triticum *inherited all alleles from tetraploid species except one with transposon insertion that most likely happened at this ploidy level (Fig. 5).

### Evidence of the *VRN1 *promoter duplication

In the present work, two and more *VRN1 *promoter copies were detected and distinguished by the presence of indel events within A genome of several spring polyploid species. There are three possible explanations of these results: (1) nonspecific duplication of different genomes, (2) heterozygous plant material with two different chromatids in one genome, (3) duplication of the investigated region. The first possibility was tested by the presence of the nucleotide substitutions and indel events specific to different genomes. For more reliable results the phylogenetic tree was constructed that demonstrated relatedness of each clone to certain genomes. Results showed an absence of nonspecificity and supported attributes of each clone to genome which amplification product was cloned. The genetic experiment and clearance of each examined lines exclude the second possible case. All accessions with two and more *VRN1 *promoter variants contained one intact promoter that was observed in winter plants. However no splitting was detected in five generations of each line.

The most likely explanation is the duplication of the investigated region that occurred in polyploid species. Previously it was supposed the duplication of the promoter region with the first exon in *Vrn-A1a *allele [10]. In most cases we found two copies of the promoter that differed by the presence of deletions. Exceptions were accessions of *T. dicoccoides *(PI428276) and cv. Jupateko of common wheat (Fig. 5).

In most polyploid *VRN1 *promoter variants, an ancestral form is an intact winter allele found only in *T. urartu *accession. The second *VRN1 *promoter sequences that was found in diploids and contained 8 bp del was inherited by some tetraploids of section *Timopheevii*. In the genome of ancestor species of this group additional 50 bp deletion occurred.

It is most likely that duplication of the promoter region occurred before deletion formation and transposon insertion. Moreover, deletions occurred in polyploid species independently from diploid species. Therefore, spring forms, which resulted from indels, were not inherited from spring diploid species. The obtained data demonstrated that quite complicated molecular alterations occurred in the studied region of spring polyploid species. Two main underlying causes of such dynamic evolutionary changes seem to be polyploidy and intensive breeding of agronomic characters.

## Conclusions

In the present study we investigated variability in the promoter region of vernalization gene *VRN1 *in spring wheat accessions. A greater variability in the *VRN1 *promoter region of polyploid species in comparison with diploid ones was observed. In diploid species, damages of *VRN1 *promoter leading to spring growth habit were inherited by cultivated forms from the wild. A complicated evolutionary process including duplication of *VRN1 *promoter was found in A genome of polyploids. Different indel events likely occurred in spring polyploid species independently from spring diploid species. Specific transposon insertions found in A genome as well as in B and G genomes of spring tetraploid species pointed to active transposition in polyploid genome. Some transcription element binding sites were observed *in silico *in the regions of indel events.

## Methods

### Plant materials

The wheat accessions, goatgrasses and rye were received from the different genebanks of Russia, Japan, the USA and ICARDA (Syria, Table 3). In the present work all used accessions were grown, and their generic definitions and growth habit (spring *vs. *winter) were determined. The botanical names of wheat species and their genomic formulas are given according to Goncharov (2002, 2009) [33,34]. *VRN1 *promoter sequences of 27 accessions belonging to four diploid wheat species (*T. urartu*, *T. boeoticum*, *T. monococcum *and *T. sinskajae*), seven goatgrass accessions belonging to *Aegilops speltoides *and *Ae. squarrosa *(syn. *Ae. tauschii*) together with 17 accessions of seven polyploid species belonging to three known sections (*Dicoccoides*, *Triticum*, *Timopheevii*) were studied. Their accession numbers, phenotypes and genotypes are given in Table 3.

### Greenhouse and genetic experiments

In the first experiment, the growth habits of di-, tetra- and hexaploid wheat species were investigated. Growth habit was analysed by spring sowing avoiding even partial vernalization. Three months after sowing in the field, when all the spring standard varieties had already headed, experimental materials were classified as spring (ear emergence) or winter (no visible ear formation).

To identify the *VRN *genotype and the number of dominant genes in diploid and tetraploid wheat segregations in the F_2 _hybrids were scored according to Goncharov (2004) [35], i.e. three months after sowing, when all the standard spring cultivars had already headed, and when it was possible to classify the hybrid plants as spring (ear emerged) or as winter (no visible ear formation). Emasculation of mother plant spikes was performed for producing F_1 _generation plants and they were pollinated by flowering father plant spikes using twirl-method. The segregation ratio for each cross was determined and compared with the expected segregation ratio using the Pierson chi-square test. Ten spring diploid accessions (*T. boeoticum *-IG116196, IG116198, K-20471, IG45296; *T. monococcum *- Mute KT3-5, *T. sinskajae *- K-48993, *Ae. squarossa*, - K-992, K-608, K-865, KU2009) and three tetraploid species (*T. turgidum *- K-16156, *T. durum *cv. Kristall, *T. turanicum *- K-15993) were studied in genetic experiments. Since allelism test was impossible between diploid species and polyploid species, only the number of dominant genes was determined for diploids.

In the second experiment we produced the near-isogenic lines based on a Black Winter Emmer *T. dicoccum *accession with dominant *Vrn *genes introgressed from near-isogenic lines of common wheat cv 'Triple Dirk' to analyse the growth habit and *VRN *genotype in tetraploid wheats [36]. They allowed to carry out methodical experiments and, later, to study allelism of dominant genes of common and tetraploid wheats.

In total, among 43 spring accessions for 10 of them the number of genes responsible for spring *vs*. winter growth habit and *Vrn *genotype for three tetraploid species were estimated in the present study, for 13 spring wheat and goatgrasses *Vrn *genotype and a number of dominant genes were known from previous works.

It was shown previously that all winter wheat had no deletions in the *VRN1 *promoter region [3,10]. The *VRN1 *promoter region of winter mPI306547 accession is available from Genbank (DQ146423). *VRN1 *promoter sequences of two other winter accessions (*T. urartu *- K-33869, *T. boeoticum *- K-25811) from our genetic experiments together with some another were investigated in the molecular part of the study for their comparison.

### Total DNA isolation, primer design, PCR amplification, cloning, DNA sequencing

The total DNA was isolated from 50-170 mg of acrospires using a standard CTAB method [37]. DNA were checked by electrophoresis in a 1% agarose gel containing ethidium bromide (0.5 mg/mL) in 1xTAE. Primer pairs used in the study are presented in the Table 4.

Selection of the appropriate oligonucleotide sequences was provided with the help of Vector NTI Suite 9.0 program [38]. All PCR reactions were performed in a volume of 20 μl containing 65 mM Tris-HCl (pH 8.9), 16 mM (NH_4 _)_2 _SO_4 _, 1.5 mM MgCl_2 _, 10% DMSO, 200 μM of each dNTP, 0.5 μМ of each primer, 20-50 ng genomic DNA template, and 1 U of Taq DNA polymerase. The touch-down PCR program had an initial strand separation step at 94°C for 3 min followed by 15 cycles of denaturation at 94°C for 30 s, annealing at 65°C with delta RT-1°C in each cycle for 30 s and elongation at 72°C for 40 s; after that, similar 25 cycles with annealing temperature 50°C. The PCR products were analyzed in agarose gel electrophoresis, extracted from gel with a Qiaquick Gel Extraction Kit (Qiagene; according to manufacturer's protocol) and either sequenced directly or cloned into pCR 4-TOPO TA vectors (Invitrogen) and then sequenced using M13 primers. 200 ng of the PCR product was used in a 10 μl cycle sequencing reaction with the ABI BigDye Terminator Kit on an ABI 377 Genetic Analyser (Applied Biosystems, http://www.appliedbiosystems.com). The obtained sequences were deposited to GenBank (Table 3).

### Allelic variation at the *VRN1 *promoter region in diploid and polyploid wheats

In the present work, *VRN1 *promoter sequences of wild and cultural diploid and polyploid wheats with different alleles were investigated to elucidate variability and possible evolution of promoter damages leading to growth habit change. PCR screening of diploid wheat was provided with primer pairs AP1_ProDel_F1/AP1_ProDel_R1 and AP1_2F/AP1_2R (Table 4). The expected PCR products were about 152 bp and 200 bp long. Using previously developed primer pairs specific for amplification of different genomes we analyzed A, B, G and D genomes of 17 tetra- and hexaploid wheats from three different sections (Table 3). Some of the PCR products were sequenced directly. Others products were cloned into a plasmid vector, and then three to five individual clones were sequenced for each sample. The length of the obtained fragments varied from about 700 bp for A genome to about 1200 bp for B genome sequences. Most of the obtained sequences contained genome-specific substitutions and indels. All sequences were deposited in the GenBank: diploids - GQ451714-GQ451749; polyploids - GQ451750-GQ451822, GQ482969-75. Spring accessions were compared with winter ones; their important differences are described below.

#### Variability of different clones inside A genome

For some polyploid accessions, PCR products of the *VRN1 *promoter region were cloned into a plasmid vector, and individual clones were sequenced. In most cases, cloning was performed for accessions where unreadable chromatograms were obtained after direct sequencing. All PCR products of studied diploids were successfully directly sequenced. Therefore, the problem could occur due to the polyploidy or the presence of different promoter variants in one genome of polyploid species [10]. It turned out that variability between different clones was sometimes quite high (Table 3). Based on the specific nucleotide substitutions, it was possible to distinguish between different genomes of polyploid species.

### Sequences and phylogenetic analysis

The nucleotide sequences were aligned using ClustalW [39] and improved by MUSCLE algorithm in UGene software http://ugene.unipro.ru edited using the GenDoc Version 2.6.002 [40]. The phylogenetic analysis was performed in the MEGA 4.0 program based on all obtained sequences of the different clones from polyploid wheat (GQ451750-93, 95-99; GQ451800-19). Sequences of the different genomes of winter *T. aestivum *TDC line (AY747600, AY747604, AY747606) were used as a control, and *vrn-H1 *sequence of *Hordeum vulgare *cv. *Strider*, as an outgroup (AY750993) [41]. Statistical support for the tree was evaluated by bootstrapping [42].

### Regulatory sites analysis

The UniPro Ugene software was used for searching and analysis of repeat and regulatory elements. The integrated plugin Sitecon was used for TF site recognition. This tool was developed for detecting conservative conformational and physicochemical properties in transcription factor binding site alignments and for site recognition [43].

## Authors' contributions

KG designed the study, carried out the molecular and bioinformatics experiment, drafted the manuscript. EYK and NPG carried out greenhouse and genetic analysis. NPG helped draft the manuscript. AB conceived the study, helped with an interpretation the results and critically revised the manuscript. All authors read and approved the final manuscript.

## Supplementary Material

Additional file 1 **Alignment of *VRN1*_*D *genome sequences**. *T. aestivum *TDC *vrn-A1, B1, D1 *were obtained from GenBank: AY747600, AY747604, AY747606. D1, D2, D3... - depict different clones of one sample. MIR 808 -Mironovskaya 808 (winter), MIR_S - Mironovskaya yarovaya (spring), MIR_W - Mironovskaya yubileinaya (winter), N5BT5D - nulli5B-tetra5D, PIR28 - Pyrothrix 28, S/V - s: Saratovskaya/Vietnamskaya 5R(5A). Click here for file

Additional file 2 **Alignment of *VRN1*_*B, G *genome sequences**. *T. aestivum *TDC *vrn-A1, B1, D1 *were obtained from GenBank: AY747600, AY747604, AY747606. B1, B2, B3...- depict different clones of one sample. MIR 808 -Mironovskaya 808 (winter), MIR_W - Mironovskaya yubileinaya (winter), SAR/VIE - s: Saratovskaya/Vietnamskaya 5R(5A). Click here for file

Additional file 3 **Maximum likelihood phylogenetic analysis of the obtained *VRN1 *clones**. *T. aestivum *TDC *vrn-A1, B1, D1 *were obtained from GenBank: AY747600, AY747604, AY747606. *vrnH1 *sequence of *Hordeum vulgare *(Strider) was used as an outgroup (Genbank: AY750993). A1, A2, A3... - depict different clones of one sample. T_d - *T. dicoccum*, T_tu - *T. turanicum*, T_aes - *T. aestivum*, Tetra - tetraploid line, T_ti - *T. timopheevii*, T_t - *T. turgidum*, T_a - *T. araraticum*. MIR 808 -Mironovskaya 808 (winter), MIR_S - Mironovskaya yarovaya (spring), MIR_W - Mironovskaya yubileinaya (winter), N5BT5D - nulli5B-tetra5D, PYR - Pyrothrix 28, S/V - s: Saratovskaya/Vietnamskaya 5R(5A), Jup - Jupateko, Os - Osijek. Sequences of tetraploid PI428276 - Genbank: GQ482969-75 Click here for file
