# Supplementary figures and images for: Molecular characterization of vernalization loci VRN1 in wild and cultivated wheats
Source: BMC Plant Biol. 2010 Aug 11;10:168. doi: 10.1186/1471-2229-10-168 (PMC3095301; doi:10.1186/1471-2229-10-168)

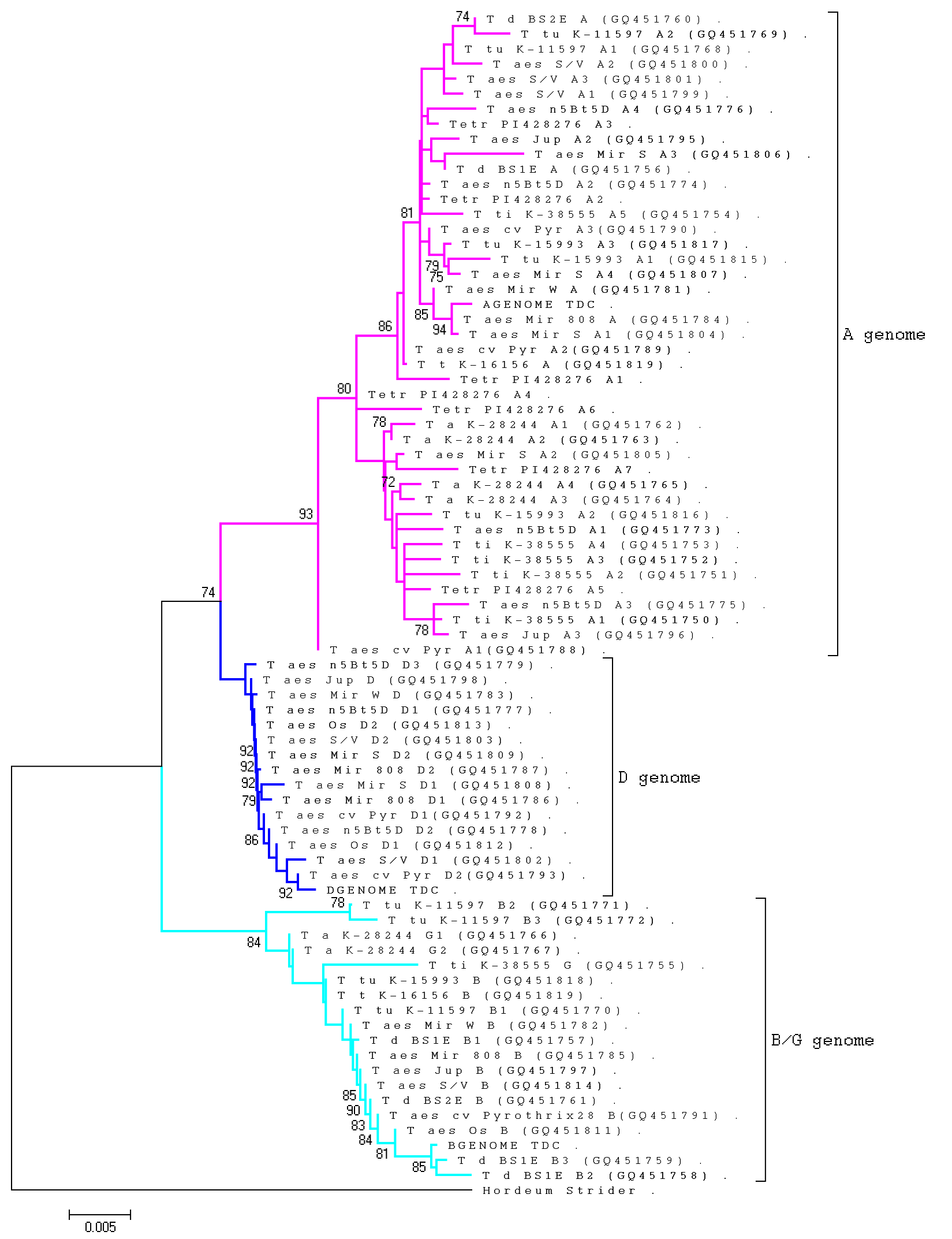

Supplement: Additional file 3 — Maximum likelihood phylogenetic analysis of the obtained VRN1 clones. T. aestivum TDC vrn-A1, B1, D1 were obtained from GenBank: AY747600, AY747604, AY747606. vrnH1 sequence of Hordeum vulgare (Strider) was used as an outgroup (Genbank: AY750993). A1, A2, A3... - depict different clones of one sample. T_d - T. dicoccum, T_tu - T. turanicum, T_aes - T. aestivum, Tetra - tetraploid line, T_ti - T. timopheevii, T_t - T. turgidum, T_a - T. araraticum. MIR 808 -Mironovskaya 808 (winter), MIR_S - Mironovskaya yarovaya (spring), MIR_W - Mironovskaya yubileinaya (winter), N5BT5D - nulli5B-tetra5D, PYR - Pyrothrix 28, S/V - s: Saratovskaya/Vietnamskaya 5R(5A), Jup - Jupateko, Os - Osijek. Sequences of tetraploid PI428276 - Genbank: GQ482969-75 [file 1471-2229-10-168-S3.TIFF]
